# Trends and mechanistic insights into hypertension-associated hearing loss: a 20-year perspective (2005–2025)

**DOI:** 10.3389/fnmol.2026.1864657

**Published:** 2026-09-08

**Authors:** Jianlong Xiong, Tao Xu, Huan Wu, Liubo Xiang, Zhihao Zhao, Yuanpeng Deng, Wansheng Wang

**Affiliations:** 1 The Second Clinical Medical College, Guizhou University of Chinese Medicine, Guiyang, China; 2 Department of Cardiovascular Internal Medicine, Second Hospital, Guizhou University of Traditional Chinese Medicine, Guiyang, China

**Keywords:** bibliometric analysis, gene target, hearing loss, hypertension, mechanism

## Abstract

**Introduction:**

Hypertension and hearing loss (HL) are increasingly prevalent in rapidly aging societies and pose major public health challenges; existing evidence suggests a potential association between the two. Although the association between hypertension and HL has received extensive attention, there is still a lack of in-depth exploration and systematic summary regarding the potential molecular interaction mechanisms underlying their coexistence, as well as the overall research trends in this field.

**Methods:**

Publications from 2005 to 2025 were retrieved from the Web of Science Core Collection, Scopus, and PubMed; 2,351 records were included and analyzed using Bibliometrix, CiteSpace, and VOSviewer. In parallel, GeneCards, GEO, and STRING were used to identify and integrate relevant molecular targets and pathways.

**Results:**

Overall publication output showed a sustained upward trend, with the United States and China contributing most prominently. Keyword and clustering analyses highlighted aging- and sex-related themes (such as “aged,” “male”) and suggested a shift from descriptive clinical observations toward etiological and therapeutic investigations. Author- and institution-level analyses indicated that Frank Robert Lin, Harvard University, and Johns Hopkins University were highly active and influential in this field. Gene intersection analysis identified 903 genes shared between hypertension and HL; IL1A, TGFB1, EGFR, MMP9, TP53, ICAM1, and BCL2 emerged as prominent network nodes. Enrichment analyses of GeneCards-derived genes and GEO-derived differentially expressed genes consistently implicated the AGE–RAGE, PI3K–Akt, and cGMP–PKG signaling pathways, fluid shear stress and atherosclerosis, as well as biological processes involving the response to oxidative stress and the regulation of blood circulation.

**Conclusion:**

These findings support incorporating hearing risk stratification and early screening into hypertension management and provide a mechanistic framework for actionable multi-target strategies, potentially enabling earlier identification and intervention and reducing subsequent treatment burden.

## 1 Introduction

With the accelerating global population aging, health challenges arising from the convergence of chronic non-communicable diseases and sensory dysfunction have become increasingly prominent; hypertension and hearing loss (HL) have emerged as two major public health concerns and are both highly prevalent among older adults (Li et al., 2025). According to the World Health Organization, in 2024 approximately 1.43 billion adults aged 30–79 years worldwide had hypertension, accounting for 33% of this age group, and nearly two-thirds lived in low- and middle-income countries (Al-Makki et al., 2022). Hypertension is not only a key risk factor for the onset and progression of cardiovascular disease (GBD 2021 ASEAN Cardiovascular Diseases Collaborators, 2025), but is also characterized by persistently elevated arterial blood pressure; if inadequately controlled over the long term, it can lead to severe and even life-threatening complications such as coronary heart disease, stroke, and renal failure (Inoue, 2025). Meanwhile, HL, a common sensory disorder in later life, can markedly impair communication and social participation; given that hypertension can cause systemic vascular injury and multi-organ target-organ damage (Goorani et al., 2024), their coexistence may further exacerbate functional limitations and increase healthcare and caregiving burdens. Therefore, systematically evaluating the association between hypertension and HL and elucidating the underlying mechanisms is of considerable importance for hearing-risk identification and early screening in hypertensive populations, as well as for developing comprehensive intervention and management strategies.

In recent years, the comorbidity of hypertension and HL and their potential association have increasingly attracted research attention. An epidemiological analysis based on data from Chinese adults collected between 2002 and 2022 showed that comorbidity patterns involving hypertension and other concurrent conditions were common. Among these patterns, the combination of hypertension and HL was the most prevalent, accounting for 10.4% (Hu et al., 2024). A case–control study likewise observed a significant association between hypertension and HL; after accounting for age and sex, hypertension remained an independent risk factor for HL and may accelerate age-related degeneration of the auditory organ (Mogi et al., 2024). Although epidemiological and cross-sectional studies have indicated an association between these conditions, the causal pathway and key biological mechanisms underlying this relationship remain incompletely understood. Noise exposure has been repeatedly used to investigate the relationship between hypertension and HL, as it may damage the cardiovascular system by altering gene networks and epigenetic pathways and by inducing oxidative stress and inflammation (Münzel et al., 2021). In addition, hemodynamic abnormalities intrinsic to hypertension may directly affect the auditory system: some studies have suggested that elevated vascular pressure may precipitate inner-ear hemorrhage via territories supplied by the anterior inferior cerebellar artery, thereby causing progressive or sudden HL, indicating that hypertension as a vascular circulatory disorder may impair auditory function through multiple mechanisms (Cavallaro et al., 2023). Hypertension is independently associated with damage to the peripheral auditory system, leading to high-frequency HL, whereas its impact on central auditory processing appears more subtle and mainly involves spatial auditory resolution (Przewoźny et al., 2016). Overall, HL is more prevalent among individuals with hypertension; however, the strength of this association is generally substantially attenuated after adjustment for age, noise exposure, diabetes, smoking, and ototoxic medication use (Jin et al., 2024). Current evidence supports considering hypertension a potential modifiable vascular risk factor or risk marker for HL. Therefore, systematically clarifying the association between hypertension and HL and identifying key actionable pathways may provide a basis for hearing-risk stratification, early assessment, and the development of integrated intervention strategies in hypertensive populations.

Despite substantial evidence suggesting an association between hypertension and HL, systematic integration of their co-evolution and interactions remains insufficient. Existing findings are prone to bias due to multiple factors, including age, sex, ototoxic medications, and noise exposure (Förster et al., 2025), and many studies still focus on identifying cross-links between “hypertension-specific mechanisms” and “HL-specific mechanisms,” leaving the overall network of reciprocal influences and coordinated regulation inadequately characterized. Against this background, we established a multidimensional research framework integrating bibliometrics, network pharmacology, and visual analytics to consolidate information at two levels: research trends and interactive molecular mechanisms. With deepening global aging and advances in molecular and genetic detection technologies, together with the continuous refinement and optimization of hearing-loss animal models (Chuan et al., 2023; Liu et al., 2023), it has become increasingly urgent to update and integrate our understanding of the comorbid mechanisms linking hypertension and HL. Notably, incorporating HL into the assessment of hypertension-related target-organ damage and exploring corresponding diagnostic and therapeutic strategies has important practical significance and translational value. However, to date, no authoritative guideline has provided explicit recommendations for the management and treatment of “hypertension comorbid with hearing loss,” and clinical practice still lacks an actionable strategy pathway. In this study, through trend identification, knowledge-map construction, and molecular network interrogation, we aim to establish a closed-loop trajectory from the evolution of the research landscape to mechanistic insights and ultimately to clinical translation, thereby providing a theoretical foundation and methodological support for developing cross-disease, multi-target intervention strategies for hypertension in the context of precision medicine.

## 2 Materials and methods

### 2.1 Data sources

**FIGURE 1:**
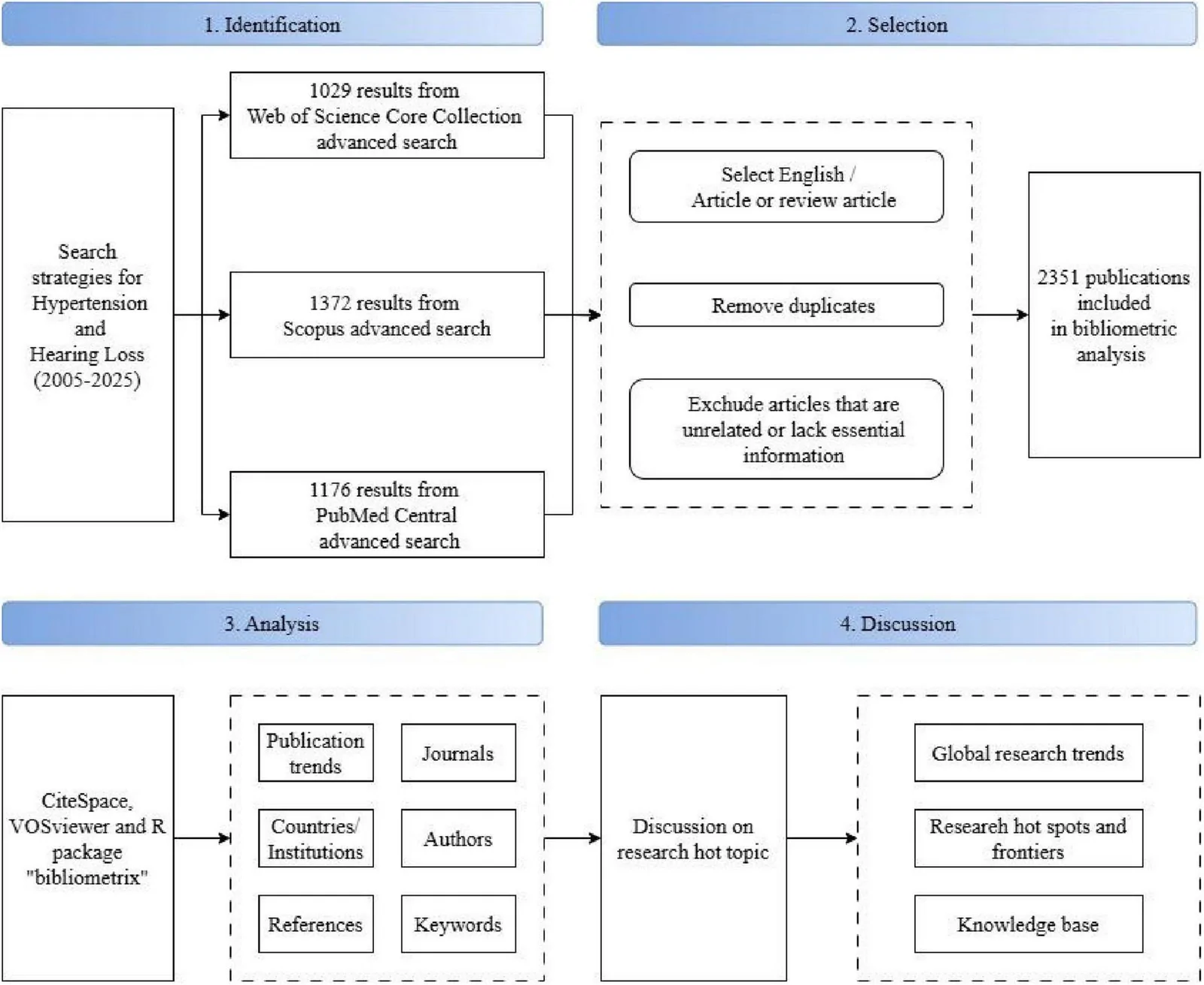
Flow diagram of study selection, including literature inclusion and exclusion.

A literature search was conducted in the Web of Science Core Collection (WoSCC), Scopus, and PubMed databases. Keyword selection was based on a broad definition of the field and related terminology, encompassing medical terms, Medical Subject Headings (MeSH) vocabulary, and disease-related synonyms. The search strategy was as follows: (“Hypertension” OR “High blood pressure” OR “Hypertensive” OR “Hyperpiesis” OR “Hypertonia” OR “Renovascular hypertensive” OR “Essential hypertension” OR “Primary hypertension” OR “Genetic hypertension” OR “Resistant hypertension” OR “Secondary hypertension” OR “Renal parenchymal hypertension” OR “Hyperpiesia”), AND (“Hearing Loss” OR “Hearing Impairment” OR “Hypoacusis” OR “Central Hearing Loss” OR “Cortical Deafness” OR “Peripheral Hearing Loss”). Searches were performed using the TS field in WoSCC, the TITLE-ABS-KEY field in Scopus, and the MeSH Terms and Title/Abstract fields in PubMed Central, with a time span from January 1, 2005 to December 31, 2025. The study selection workflow (Figure 1) was conducted independently by two researchers; disagreements were resolved by a third researcher who participated in adjudication and validated the results. To ensure the scientific quality and consistency of the included literature, only English-language original research articles and review articles were retained for analysis. Under this search protocol, 1,029 records from WoSCC, 1,372 from Scopus, and 1,176 from PubMed were collected and downloaded. After applying the screening criteria and removing duplicates, 2,351 eligible publications were ultimately included for bibliometric analysis and molecular mechanism exploration of the association between hypertension and HL.

### 2.2 Bibliometric analysis

To systematically explore research trends and potential interaction mechanisms between hypertension and HL, a series of bibliometric and bioinformatics tools were employed. Owing to its multidimensional analytical capabilities and highly interactive visualizations, the R-based Bibliometrix package (version 5.0) can effectively delineate research landscapes and association patterns, providing key support for domain-level analyses (Ozen Cinar, 2025). In addition, CiteSpace (6.2.R4) and VOSviewer (1.6.20) were used for bibliometric analyses. Quantitative indicators, including publication output and collaboration patterns, were computed using the bibliomeReader R package. VOSviewer was used to further characterize co-authorship networks and keyword co-occurrence relationships, and Scimago Graphica (1.0.40) was used to enhance the spatial visualization of statistical relationships. CiteSpace was applied for mapping and clustering analyses of countries, institutions, journals, and keywords. The parameter settings were as follows: the time span was set to 2005–2025 with a 1-year time slice and the number of breakpoints set to 4; institutions and keywords were specified as node types. The clustering algorithm was set to “Walktrap,” while all other parameters were kept at their default values. The *k*-value was set to 25, and keyword clustering was performed using the LLR algorithm.

### 2.3 Disease mechanism exploration

The GSE7414 and GSE284387 datasets were obtained from the Gene Expression Omnibus database.^1^ These datasets were published by Rouet P et al. and Fan G et al., respectively. GSE7414 contains differentially expressed genes between the hypertension and healthy control groups, whereas GSE284387 contains transcriptomic alterations associated with HL. Disease-related mRNA expression profile data were extracted from these datasets. The goodSamplesGenes function was used to remove outliers, and the top 50 differentially expressed genes ranked according to the median absolute deviation were selected for subsequent enrichment analysis to identify shared signaling pathways.

For molecular mechanism exploration, GeneCards^2^ was used to screen potential gene targets associated with hypertension and HL, and genes with relevance scores greater than 5 were selected as potential target genes. Corresponding target screening was also performed for the HL subtypes peripheral hearing loss (PHL) and central hearing loss (CHL). Based on the overlap of disease-associated genes, the STRING database was further used to construct a protein–protein interaction (PPI) network of shared genes. Cytoscape (v3.9.1) was used for visualization to identify core network modules and hub genes, thereby delineating the functional clustering architecture within each subtype.

Enrichment analyses were performed on these gene targets to elucidate their roles in biological processes, molecular functions, and disease pathways. Multiple R packages, including “clusterProfiler,” “enrichplot,” “ggplot2,” “DOSE,” and “ComplexHeatmap,” were used to perform Disease Ontology (DO), Kyoto Encyclopedia of Genes and Genomes (KEGG), and Gene Ontology (GO) enrichment analyses, thereby identifying subtype-specific molecular mechanisms and biological processes associated with different HL subtypes.

## 3 Results

### 3.1 General analysis

**FIGURE 2:**
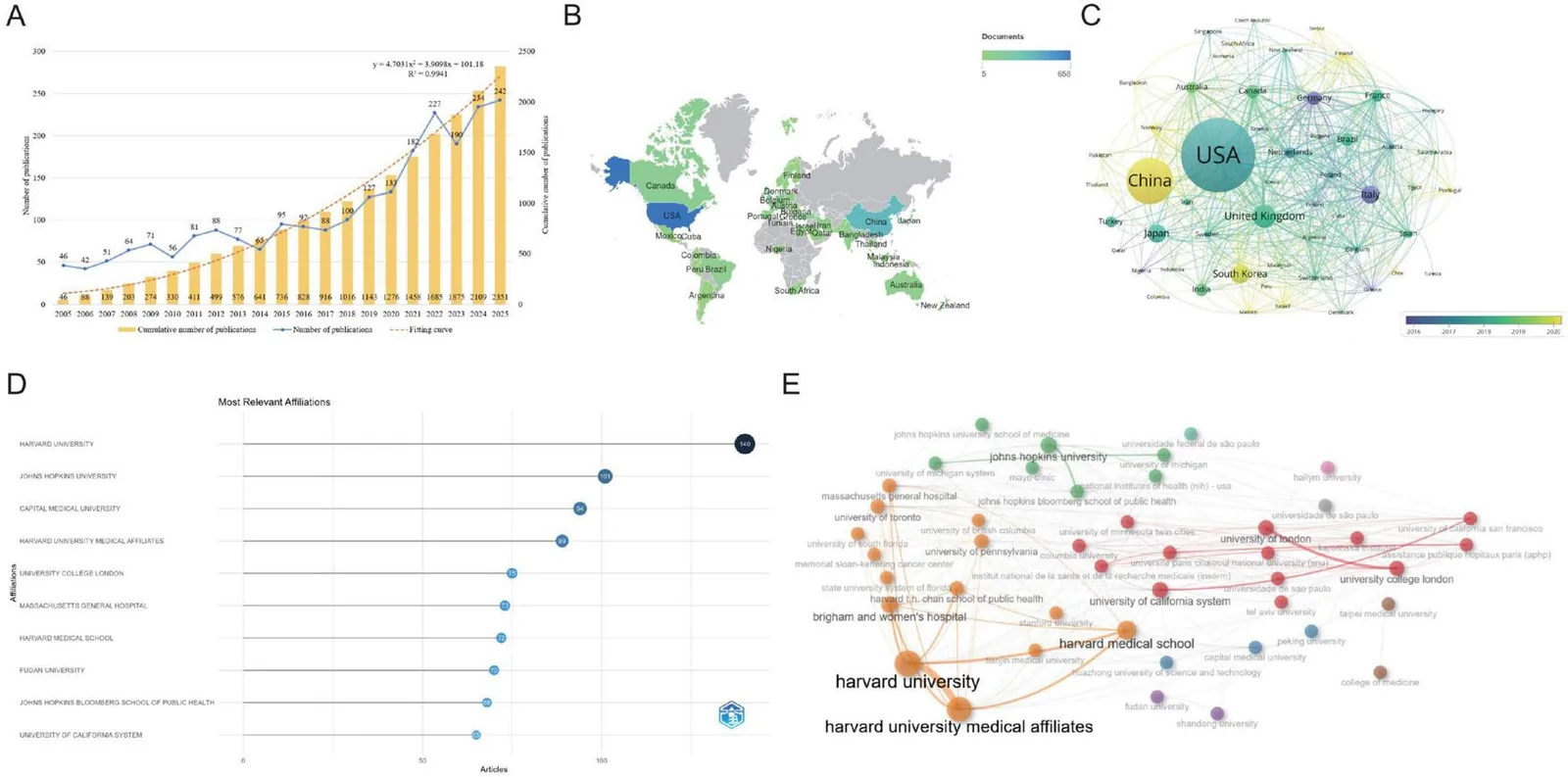
Overall trends and knowledge mapping of hypertension–HL research: publication output, collaboration networks, and institutional contributions. **(A)** Annual and cumulative publication counts. **(B)** Geographic distribution of publications by country. **(C)** Country co-occurrence (collaboration) network. **(D)** Top 10 institutions by publication output. **(E)** Institutional co-occurrence (collaboration) network.

Over the past 21 years, a total of 2,351 publications related to hypertension and HL were retrieved from the WoSCC, Scopus, and PubMed databases (Figure 2). The publication trend from 2005 to 2025 exhibited three distinct phases: a slow increase from 2005 to 2017, a sharp increase from 2018 to 2022, and a return to a stable growth trend from 2023 to 2025, maintaining a high level of annual output. Notably, the annual number of publications related to hypertension and HL remained at a peak in 2025, indicating substantial attention to this field in recent years. Publication trends were analyzed using Microsoft Excel 2024, and the results showed a strong correlation between cumulative publication output and year (Figure 2A). Collectively, these findings indicate that an integrated multi-database analytical strategy can provide a more comprehensive data foundation for constructing a domain knowledge map and elucidating its evolutionary trajectory.

### 3.2 Distribution of countries, institutions, authors, journals, and references

In terms of international collaborative output (Figure 2B), the top 10 countries contributed 1,893 publications in total, with the United States producing the largest number of relevant publications (*n* = 658), followed by China (*n* = 374), and the United Kingdom ranking third (*n* = 162) (Table 1). Figure 2C visualized collaboration relationships among countries using VOSviewer; publications from the USA, China, and the UK showed relatively high quality, which may be attributable to their close collaboration with other countries and institutions.

**TABLE 1:** Top 10 productive countries and institutions concerning hypertension and hearing loss research.

Rank	Country	Articles	Affiliation	Articles
1	USA	658	Harvard University	140
2	China	374	Johns Hopkins University	101
3	UK	162	Capital Medical University	94
4	South Korea	127	Harvard University Medical Affiliates	89
5	Japan	123	University College London	75
6	Italy	120	Massachusetts General Hospital	73
7	Germany	85	Harvard Medical School	72
8	Canada	83	Fudan University	70
9	India	81	Johns Hopkins Bloomberg School of Public Health	68
10	Brazil	80	University of California System	65

Table 1 lists the top 10 institutions by publication output. Harvard University ranked first with 140 publications, and Johns Hopkins University ranked second (*n* = 101) (Figure 2D). Figure 2E, generated using CiteSpace, presents the inter-institutional collaboration network. These maps indicate that a relatively dense collaborative structure has been formed among institutions, reflecting close external collaboration in this field. Analysis of these collaboration networks can effectively identify cross-domain collaboration opportunities, optimize the allocation of research resources, and ultimately catalyze research innovation.

**FIGURE 3:**
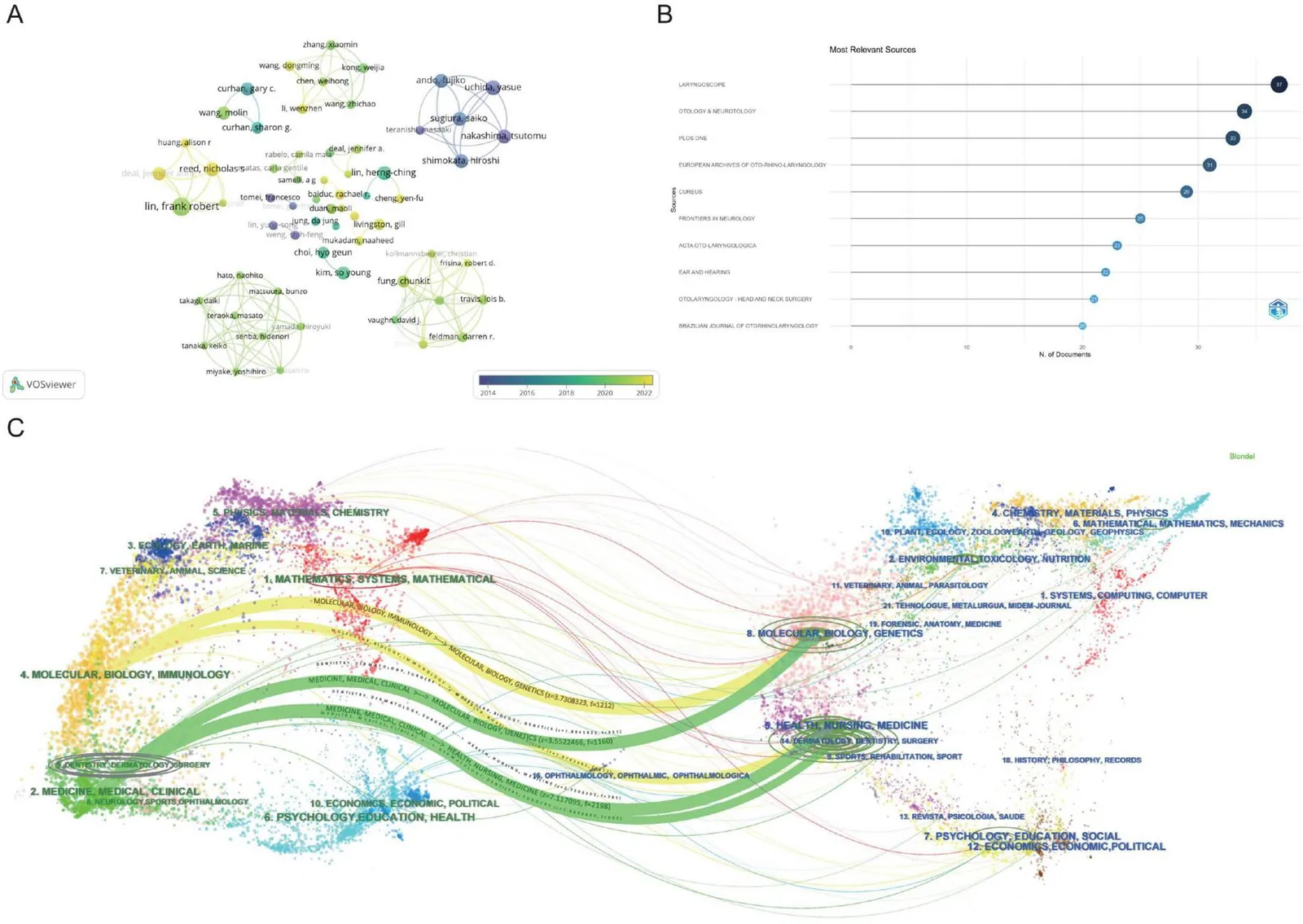
Author collaboration and journal distribution of publications on hypertension and HL from 2005 to 2025. **(A)** Co-authorship (collaboration) network. **(B)** Top 10 journals by publication output. **(C)** Bimap of journals publishing hypertension–HL literature.

Table 2 lists the top 10 authors by number of publications related to hypertension comorbid with HL. Over the past 25 years, Lin, Frank Robert was the most productive author, with a cumulative total of 16 publications, indicating substantial research contributions to this field. Meanwhile, Uchida, Yasue ranked second, with 12 publications related to hypertension and HL. Figure 3A shows a close collaboration network among researchers, suggesting that this research direction has attracted attention from scholars across countries.

**TABLE 2:** Top 10 popular authors in this field.

Rank	Author	Documents	Institution	Country
1	Lin, Frank Robert	16	Johns Hopkins Bloomberg School of Public Health	USA
2	Uchida, Yasue	12	National Center for Geriatrics and Gerontology	Japan
3	Ando, Fujiko	11	National Center for Geriatrics and Gerontology	Japan
4	Lin, Herng-Ching	11	Taipei Medical University	Taiwan, China
5	Nakashima, Tsutomu	11	Ichinomiya Medical Treatment and Habilitation Center	Japan
6	Reed, Nicholas S	11	Johns Hopkins Bloomberg School of Public Health	USA
7	Shimokata, Hiroshi	11	National Institute for Longevity Sciences	Japan
8	Sugiura, Saiko	11	Nagoya University School of Medicine	Japan
9	Curhan, Gary C.	10	Brigham and Women’s Hospital and Harvard Medical School	USA
10	Deal, Jennifer Anne	10	Johns Hopkins Bloomberg School of Public Health	USA

Figure 3B presents the top 10 journals by publication output in this field. The Laryngoscope ranked first with 37 publications, indicating the journal’s strong support for advances related to hypertension comorbid with HL. Figure 3C shows the overlap among journals and illustrates the thematic distribution. The map indicates that research outputs in molecular biology, genetics, and immunology exhibit pronounced interdisciplinary characteristics and have been widely adopted and cited in journals across multiple related fields, including dermatology, surgery, and dentistry. Table 3 presents the top 10 most-cited articles, which mainly focus on three core dimensions: (1) a multisystem, cross-disciplinary, and complex pathophysiological interaction network involving hypertension and HL; (2) shared environmental and lifestyle risk exposures; and (3) the value of integrated management and global health implications highlighted in older populations.

**TABLE 3:** The top 10 articles with the highest citation count.

Rank	Title	Year	Journal	First Author	Institution
1	Auditory and non-auditory effects of noise on health	2014	LANCET	Mathias Basner	University of Pennsylvania Perelman School of Medicine
2	Hearing loss and incident dementia	2011	ARCH NEUROL-CHICAGO	Frank R Lin	The Johns Hopkins School of Medicine
3	Prevalence and characteristics of tinnitus among US adults	2010	AM J MED	Josef Shargorodsky	Massachusetts Eye and Ear Infirmary
4	Characterizing the Pattern of Anomalies in Congenital Zika Syndrome for Pediatric Clinicians	2017	JAMA PEDIATR	Cynthia A Moore	National Center on Birth Defects and Developmental Disabilities, Centers for Disease Control and Prevention, Atlanta, Georgia.
5	GATA2 deficiency: a protean disorder of hematopoiesis, lymphatics, and immunity	2014	BLOOD	Michael A Spinner	Laboratory of Clinical Infectious Diseases, National Institute of Allergy and Infectious Diseases, Bethesda, MD
6	Population attributable fractions for risk factors for dementia in low-income and middle-income countries: an analysis using cross-sectional survey data	2019	LANCET GLOB HEALTH	Naaheed Mukadam	University College London
7	Occupational noise exposure and hearing: a systematic review	2016	INT ARCH OCC ENV HEA	Arve Lie	National Institute of Occupational Health, Oslo, Norway.
8	Chronic diseases and risk for depression in old age: a meta-analysis of published literature	2010	AGEING RES REV	Chang-Quan Huang	Sichuan University
9	Health and disease in 85 year olds: baseline findings from the Newcastle 85+ cohort study	2009	BRIT MED J	Joanna Collerton	Newcastle University
10	Long-term exposure to road traffic noise and myocardial infarction	2009	EPIDEMIOLOGY	Jenny Selander	Stockholm University

### 3.3 Keyword analysis

**FIGURE 4:**
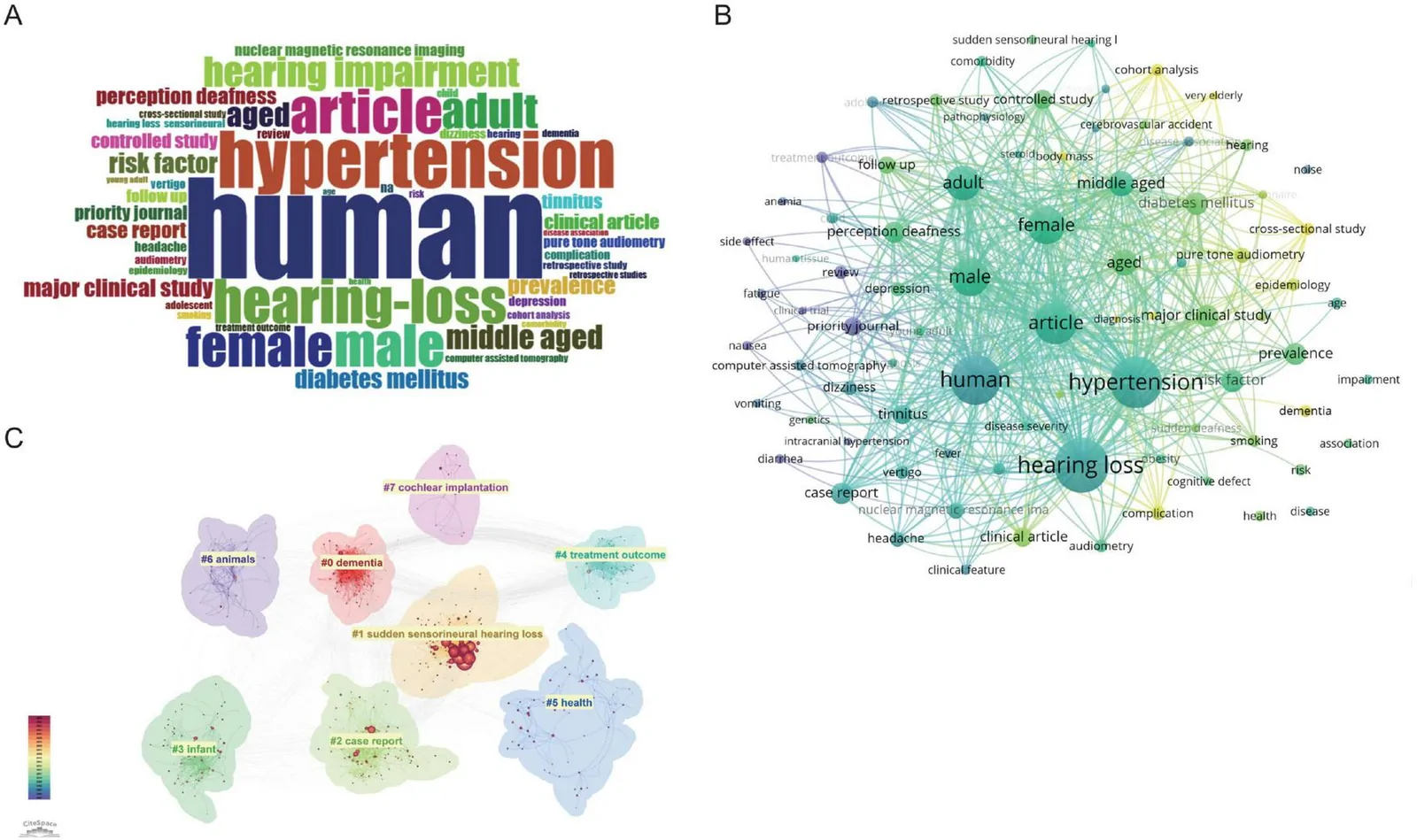
Co-citation analysis of keywords and clusters. **(A)** Burst detection of co-cited keywords. **(B)** Keyword co-occurrence network. **(C)** Major clusters in the co-citation network.

Keyword analysis provides a core methodological basis for identifying research hotspots and probing academic frontiers (Han et al., 2023). By systematically organizing the core terms and their associative structures in hypertension–HL research, this analysis effectively delineates the knowledge framework and developmental trends of this interdisciplinary field. Commonly occurring keywords in this field include “hypertension,” “article,” “hearing-loss,” and “male” (Figure 4A), and these keywords collectively form a closely interconnected thematic network (Figure 4B). Among them, terms such as “aged,” “male,” “hypertension,” and “hearing loss” reveal strong associations with aging and sex in this field, indicating that, as population aging progresses, the interaction between hypertension and HL remains a sustained research focus. Sex is also a key factor; sex differences may lead to biased effect estimates, which is consistent with some previous findings (Colafella and Denton, 2018). In addition, keywords such as “major clinical study,” “case report,” and “cross-sectional study” suggest that current research on the hypertension-HL comorbidity largely remains at the level of clinical phenomenology, with relatively limited in-depth exploration of molecular mechanisms.

Cluster analysis of keyword co-occurrence further revealed the structural composition of the research themes. The network exhibited a well-defined and convincing clustering structure, with Modularity *Q* = 0.4655 and Mean Silhouette = 0.7801, indicating clearly delineated clusters, stable research themes, and a high degree of reliability. Three broad categories of research themes were identified in this field (Figure 4C). The first category comprised #0 dementia, #1 sudden sensorineural hearing loss, #3 infant, and #5 health, primarily involving research related to diseases and clinical stages. The second category comprised #4 treatment outcome and #7 cochlear implantation, representing research directions focused on treatment and rehabilitation approaches. The third category comprised #6 animals and #2 case report, primarily focusing on basic research and therapeutic efficacy indicators.

**FIGURE 5:**
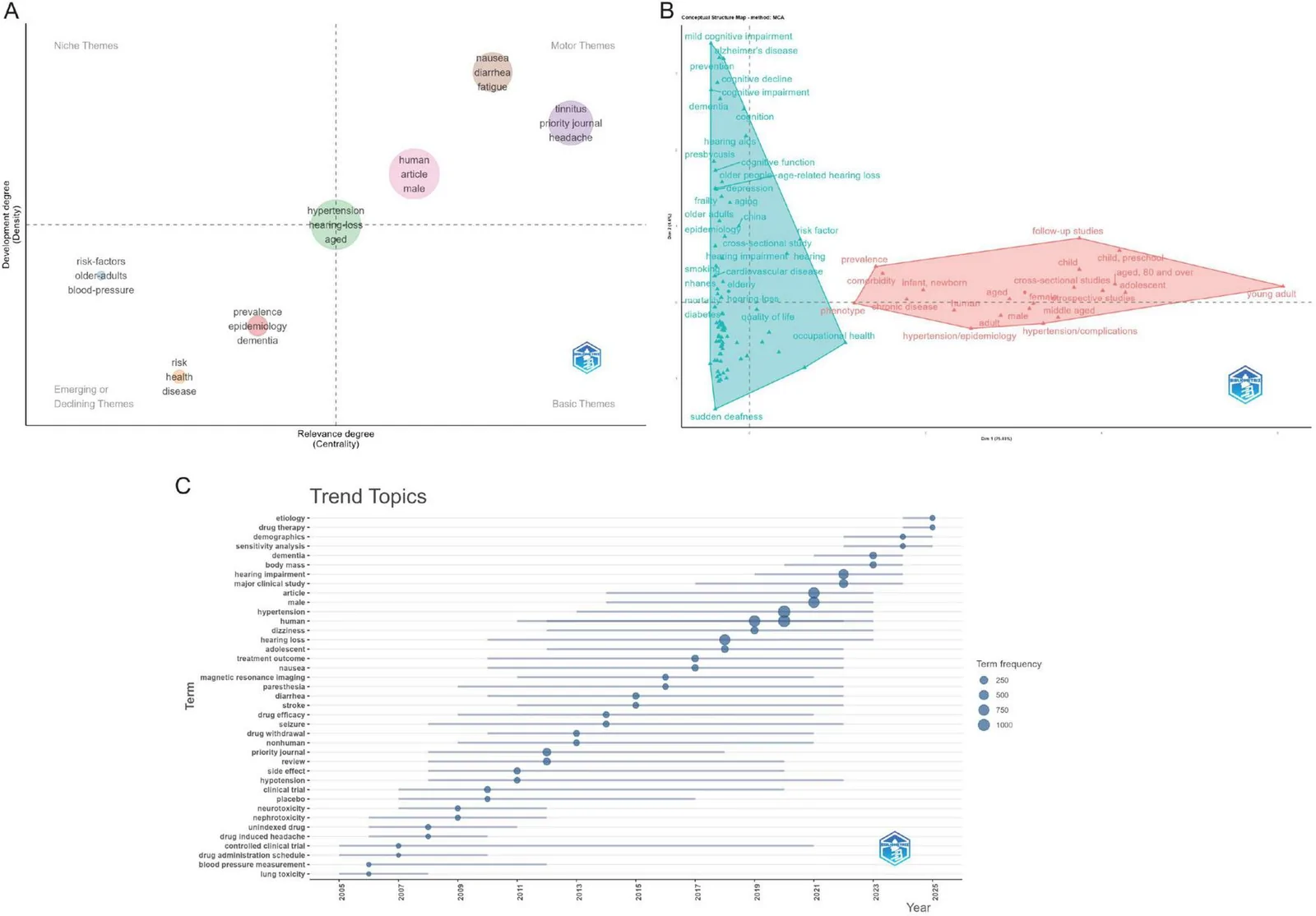
Thematic and conceptual analyses of hypertension–HL research. **(A)** Thematic strategy map (*x*-axis: centrality; *y*-axis: density); Quadrant I indicates mature and core themes, Quadrant II indicates peripheral yet well-developed themes, Quadrant III indicates emerging or declining themes, and Quadrant IV indicates basic themes with cross-domain supporting roles. **(B)** Visualization of the keyword conceptual structure. **(C)** Trend analysis of keyword-related themes.

A thematic map divided research topics into four quadrants, in which contemporary hotspots included “nausea,” “diarrhea,” “fatigue,” “tinnitus,” and “headache,” potentially indicating in-depth exploration of their underlying pathophysiological mechanisms and clinical consequences, thereby representing current hotspots. In contrast, early themes such as “risk factors,” “prevalence,” and “epidemiology” have gradually declined in importance (Figure 5A). In the conceptual structure map constructed based on Keyword Plus, the knowledge architecture of this research field was revealed, and the intrinsic connections among core concepts were visualized (Figure 5B). Thematic trend analysis yielded similar results: keywords such as “human,” “hypertension,” “hearing loss,” and “male” have increased substantially since 2018, reflecting a deepening and translational research stage that focuses on human clinical populations, aims to elucidate specific pathophysiological mechanisms, and explores precision diagnostic and therapeutic strategies (Figure 5C). Notably, current research trends have shifted toward “etiology” and “drug therapy,” indicating an increasing emphasis on studies of pathogenesis and pharmacological interventions.

**FIGURE 6:**
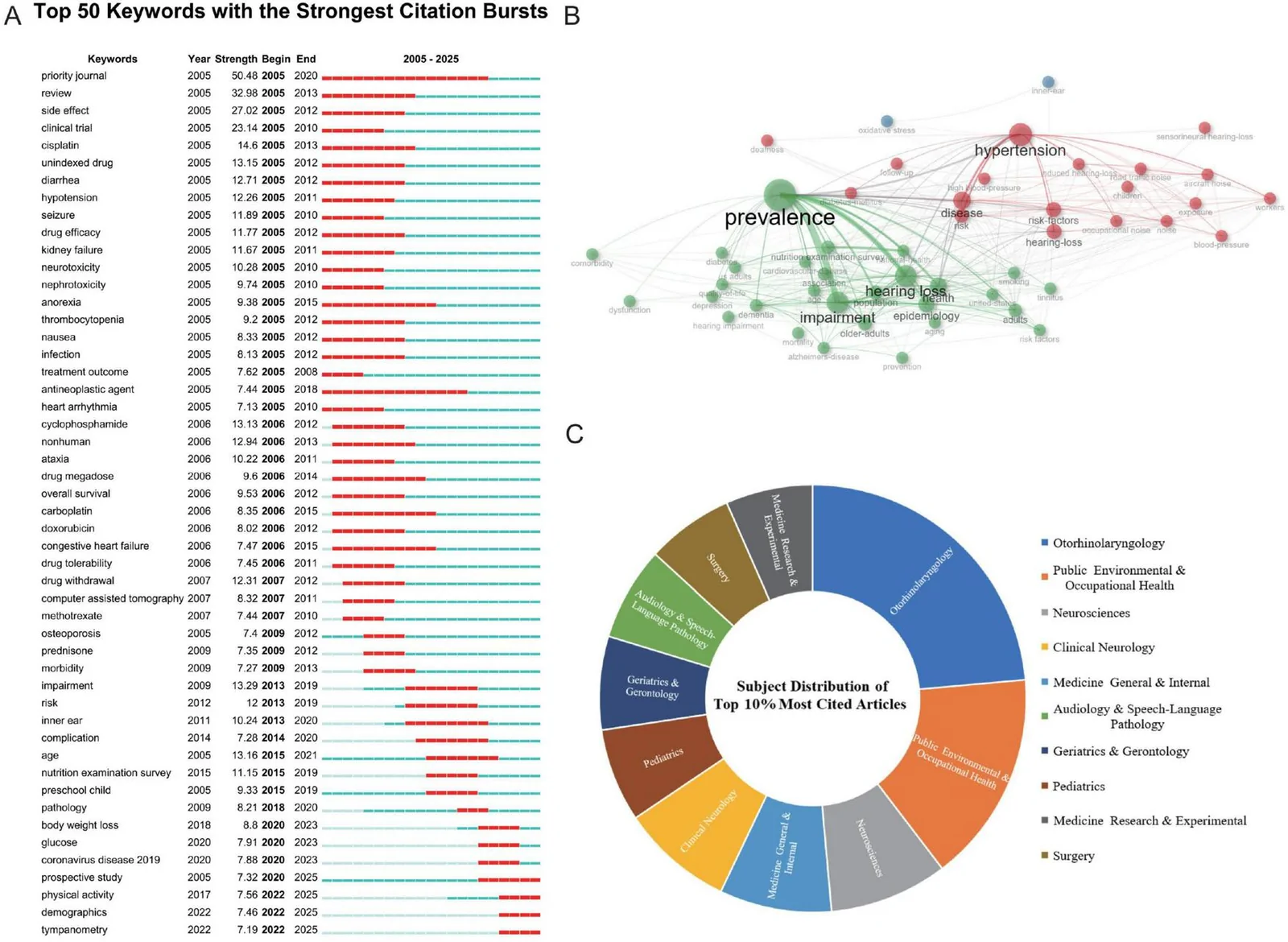
Keyword burst strength analysis and thematic distribution of the top 10% most-cited articles. **(A)** Top 50 keywords ranked by burst strength and duration. **(B)** Keyword co-occurrence network analysis of the top 10% most-cited articles. **(C)** Thematic mapping of the top 10% most-cited articles.

Keyword burst analysis assessed bibliometric terms from 2005 to 2025 based on burst strength and duration, identifying key burst terms with high intensity and sustained duration during this period. Across burst keywords, research topics underwent an evident transition: from an early focus on disease-related therapeutic approaches such as clinical trial, drug efficacy, and computer assisted tomography (2005–2018), to risk, complication, and age elucidating the increasing disease burden with age (2019–2023), and then to recent studies on more systematic and refined diagnostic and therapeutic approaches such as prospective study, physical activity, demographics, and tympanometry (2023–2025) (Figure 6A). Moreover, the thematic distribution of the top 10% most-cited articles (i.e., highly cited papers) revealed the core trends and value orientation of the field. Keyword co-occurrence network analysis identified three major clusters: the red cluster focused on the complex interrelationships between hypertension and HL, including the interaction between vascular pressure and hearing decline; the green cluster was disease-oriented, examining the relationship between specific HL and age as well as clinical quality-of-life assessment and management; and the blue cluster focused on oxidative stress and organismal mechanisms. Together, these clusters reflect the structural characteristics of the hypertension–HL research field and provide directions for future multidisciplinary studies (Figure 6B). The highly cited papers were mainly related to “Otorhinolaryngology,” “Public, Environmental and Occupational Health,” “Neurosciences,” and “Clinical Neurology,” among others (Figure 6C).

### 3.4 Genetic intersection analysis and molecular mechanism investigation of hypertension and HL

**FIGURE 7:**
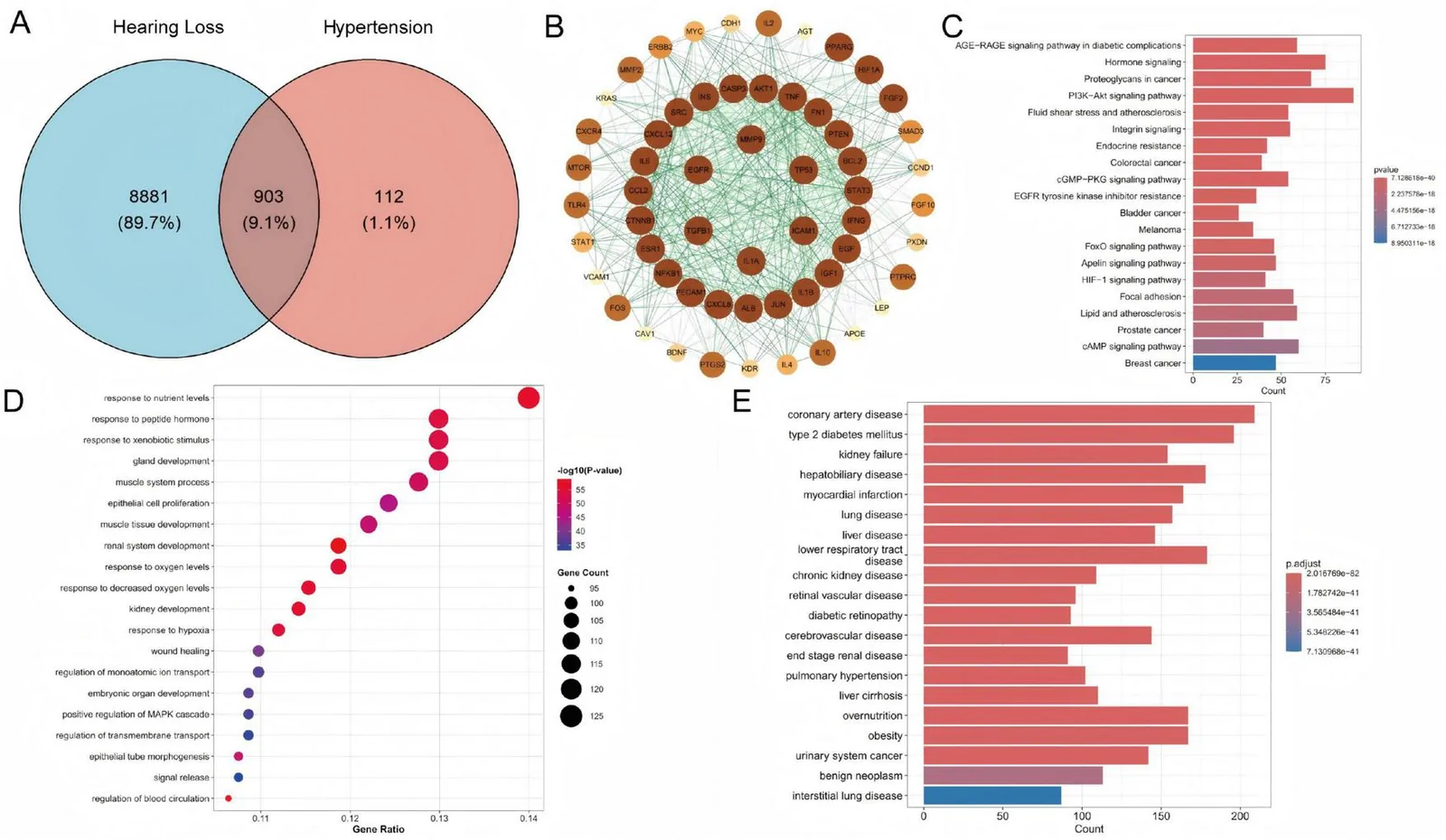
Intersection analysis of genes shared between hypertension and HL. **(A)** Intersection analysis of disease-associated genes. **(B)** Threshold analysis of intersecting genes. **(C)** KEGG enrichment analysis of intersecting genes. **(D)** GO enrichment analysis of intersecting genes. **(E)** DO enrichment analysis of intersecting genes. KEGG, Kyoto Encyclopedia of Genes and Genomes; GO, Gene Ontology; DO, Disease Ontology.

Through genetic intersection analysis, 903 shared genes were identified between hypertension and HL, including SLC26A4, CDH23, MYH9, MYH14, and ATP6V1B1 (Figure 7A). The results indicated that genes such as IL1A, TGFB1, EGFR, MMP9, TP53, ICAM1, and BCL2 play important roles in the comorbid mechanism of hypertension and HL; their interactions collectively constitute a key molecular regulatory network (Figure 7B). KEGG enrichment analysis showed that the shared genes were significantly enriched in the AGE-RAGE signaling pathway associated with metabolism-related oxidative stress and inflammation, the PI3K-Akt signaling pathway regulating cellular proliferation, metabolism, and angiogenesis, the Fluid shear stress pathway related to hemodynamics, and the cGMP-PKG signaling pathway mediating vasodilation (Figure 7C). GO analysis indicated that these shared genes play important roles in oxygen level and hypoxic stress responses, vascular endothelial morphology and muscle development processes, and the regulation of blood circulation (Figure 7D). At the Disease Ontology level, DO analysis further confirmed associations between these shared genes and cardiovascular and cerebrovascular diseases, metabolic diseases, kidney diseases, and liver and lung diseases with fibrotic features (Figure 7E).

**FIGURE 8:**
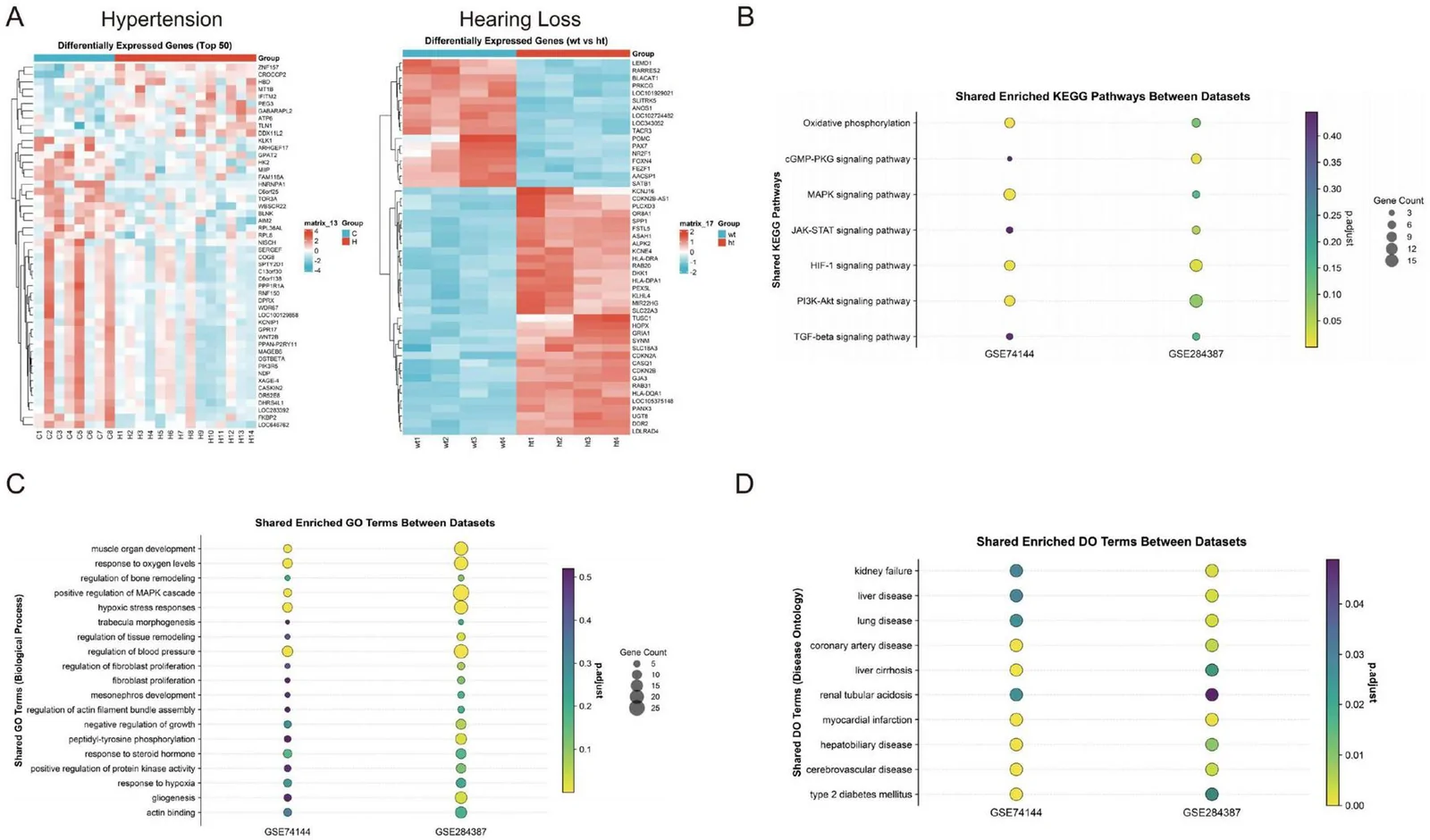
Analysis of significantly enriched terms shared by differentially expressed genes associated with hypertension and HL in the GEO database. **(A)** Top 50 differentially expressed genes in hypertension and HL. **(B)** KEGG pathways significantly enriched in both diseases. **(C)** GO terms significantly enriched in both diseases. **(D)** DO terms significantly enriched in both diseases.

In parallel, two datasets, GSE7414 and GSE284387, were selected from the GEO database. From these datasets, 62 upregulated and 121 downregulated genes were identified for hypertension, whereas 237 upregulated and 177 downregulated genes were identified for HL. Figure 8A presents the top 50 differentially expressed genes for each disease. Subsequently, 183 differentially expressed genes associated with hypertension and 414 differentially expressed genes associated with HL were subjected to separate enrichment analyses (Supplementary Figure 1), after which the significantly enriched pathways shared by both diseases were extracted. This intersection analysis at the functional module level is more robust than a simple gene-level intersection and provides a deeper, more comprehensive, and highly biologically interpretable characterization of the potential shared pathogenic mechanisms underlying the two diseases (Peng et al., 2026). Intersection of the KEGG and GO enrichment results further identified the relevant biological pathways and functions, including oxidative phosphorylation-related pathways, the cGMP-PKG signaling pathway, the PI3K-Akt signaling pathway, and biological processes associated with blood pressure regulation, response to hypoxia, and response to oxygen levels (Figures 8B, C). DO analysis indicated that both diseases were closely associated with multiple other conditions, including urinary system diseases, cardiovascular and cerebrovascular diseases, and metabolism-related diseases (Figure 8D). Intersection analysis of the gene enrichment results obtained from independent datasets further supported the associations of both diseases with the aforementioned signaling pathways.

### 3.5 Genetic intersection analysis of hypertension with HL-related subtypes PHL and CHL

In addition, a systematic interaction analysis between peripheral and central HL and hypertension revealed that PHL shared 933 intersecting genes with hypertension (Supplementary Figure 2A). Further network analysis indicated that genes such as IGF1, EGFR, MMP9, TP53, BCL2, and EGF serve as key bridging factors within the molecular network linking the two diseases (Supplementary Figure 2B). This association appears to be mainly co-mediated by signaling pathways including AGE-RAGE, PI3K-Akt, and Fluid shear stress and atherosclerosis, which regulate key pathological processes such as oxidative stress and chronic inflammation, hemodynamics, and cellular proliferation and metabolism (Supplementary Figure 2C). GO analysis suggested that the corresponding gene set plays key roles in hypoxic stress responses, responses to peptide hormone and humoral signaling, and regulation of blood circulation (Supplementary Figure 2D). DO enrichment analysis indicated that these intersecting genes are associated with multiple diseases, including cardiovascular and cerebrovascular diseases, metabolic diseases, kidney diseases, and retinal microvascular lesions (Supplementary Figure 2E).

**FIGURE 9:**
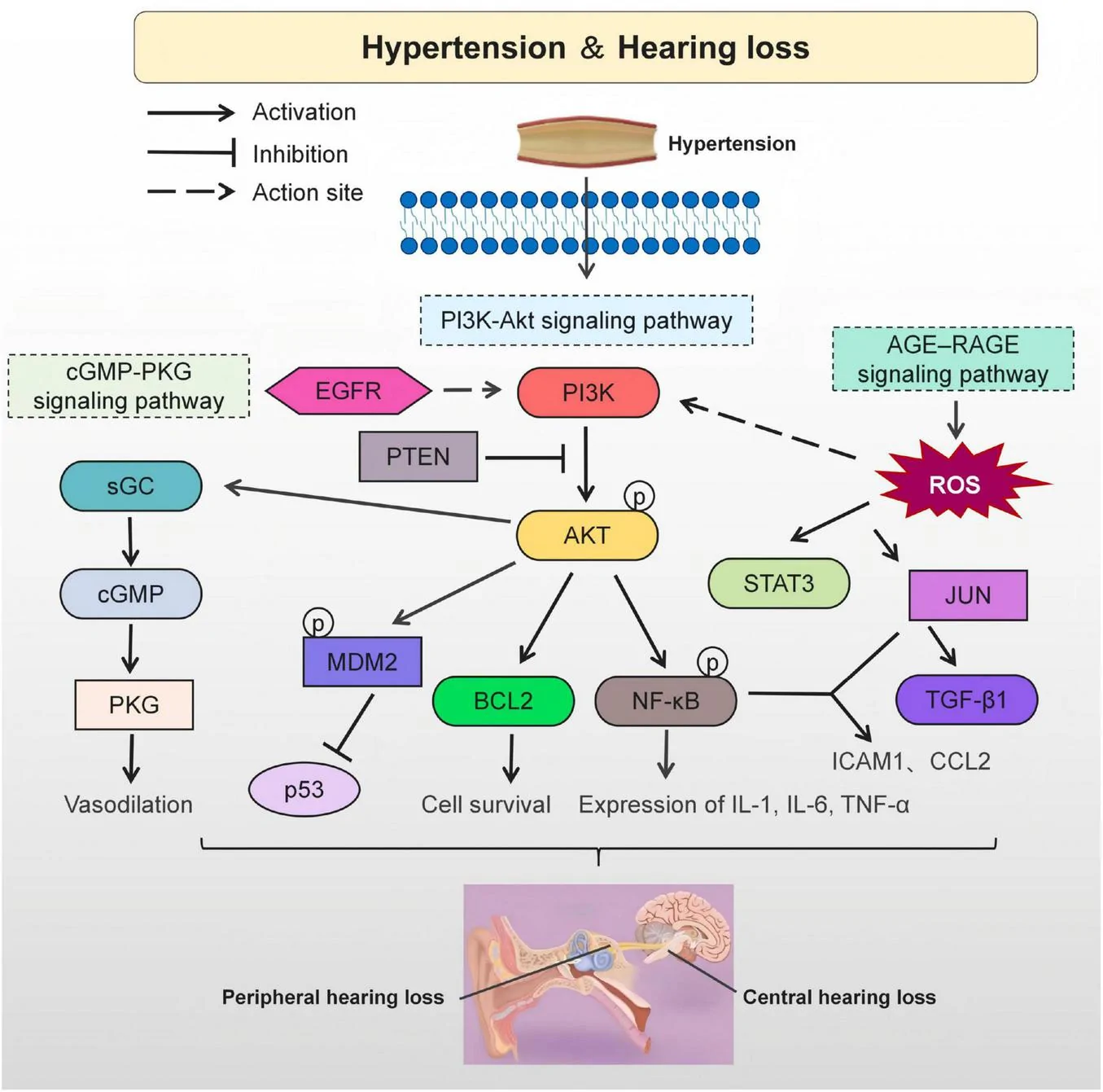
Potential relationship between hypertension and HL.

A total of 951 intersecting genes were identified between CHL and hypertension (Supplementary Figure 3A). Genes such as CXCL12, EGFR, TP53, MMP9, BCL2, and FGF2 were located at hub positions in the disease interaction network (Supplementary Figure 3B) and were significantly enriched in pathways including AGE-RAGE, PI3K-Akt, and cGMP-PKG (Supplementary Figure 3C). GO analysis showed that the biological characteristics of this gene set were concentrated in hypoxic stress responses, regulation of blood circulation, responses to peptide hormone signaling, injury repair and inflammatory responses, and muscle tissue development, with certain mechanistic differences compared with PHL (Supplementary Figure 3D). Further DO analysis revealed that these intersecting genes were likewise closely associated with cardiovascular and cerebrovascular diseases, metabolic diseases, kidney diseases, and retinal microvascular lesions (Supplementary Figure 3E). In addition, based on the significant interaction and comorbidity between hypertension and HL, as well as their multiple shared features in pathogenesis, and pathological progression (Figure 9), this study systematically delineated the genetic association landscape between the two conditions and subsequently proposed an integrative synergistic intervention strategy.

## 4 Discussion

Hypertension, one of the most prevalent chronic cardiovascular diseases worldwide, shows a marked age-dependent increase in prevalence and has become an important health issue in older adults. Previous studies have demonstrated a clear and significant association between hypertension and HL; the two conditions frequently coexist in older populations and may interact bidirectionally. Unaddressed HL not only impairs speech understanding and communication, but also reduces social participation, exacerbates loneliness, and may increase the risk of depression and all-cause mortality in older adults (Bao et al., 2019; Völter et al., 2020); these adverse consequences may further aggravate blood pressure variability, forming a vicious cycle of psychosomatic interaction. Existing evidence also suggests that multiple hypertension-related indices are independently associated with the risk of HL and may serve as potential predictors (Hou and Liu, 2024; Zhang et al., 2024; Huang et al., 2025). Therefore, early identification and screening for hearing risk in older adults with hypertension, optimization of relevant predictive indicators, and the implementation of targeted, standardized intervention strategies may help prevent further progression of HL.

In this study, we integrated resources from the WoSCC, Scopus, and PubMed to establish a complementary literature-analytic framework, thereby broadening literature coverage and enhancing the depth of knowledge-network interrogation. Using bibliometric analyses in conjunction with genetic intersection analyses, we systematically reviewed research hotspots, thematic evolution, potential disease–disease interactions, and shifts in putative molecular mechanisms in this field over the period 2005–2025. The results indicate that research on hypertension comorbid with HL is in a pivotal developmental stage characterized by concurrent growth in scholarly output and influence, exhibiting a persistently active evolutionary trajectory. The annual publication growth pattern from 2005 to 2025 suggests a critical transition from early consensus formation toward accelerated mechanistic deepening and clinical translational application; notably, a marked increase occurred in 2022 compared with the previous year, with high levels maintained in 2024–2025, reflecting accumulating evidence for the hypertension-HL association and broadening interdisciplinary attention across cardiovascular medicine, otorhinolaryngology, and geriatrics.

From the perspective of national (or regional) and institutional publication patterns, the United States remains dominant in publication output, while countries such as China and the United Kingdom also show upward trends in productivity. Notably, at the level of international collaboration, core knowledge production, methodological paradigms, and frontier agenda setting in this field are, to some extent, concentrated within the central academic networks of a small number of countries, exhibiting a relatively distinct “core–periphery” structure. Although the United States is the most active in international collaboration, the number of its coauthored publications remains substantially lower than that of its independently authored publications, suggesting that international collaboration constitutes a relatively limited and potentially more selective proportion of its total output; for other partners in the collaboration network, this pattern may imply a degree of asymmetric dependence and may attenuate the consideration of disease heterogeneity across regions, ethnicities, and healthcare systems in research question formulation. Accordingly, future international collaboration should further promote equitable scientific dialogue and joint agenda setting to reduce potential geographic bias and facilitate a more balanced knowledge ecosystem. At the author level, over the past 21 years, scholars such as Lin, Frank Robert and Uchida, Yasue have constituted an important academic force in this field, contributing key research outputs and serving as active nodes within collaboration networks, exerting influence beyond individual publications through methodological dissemination and talent aggregation.

With accelerating population aging, the incidence of age-related HL is increasing substantially. In older adults, vascular functional decline and aging-related multisystem deterioration, compounded by environmental exposures and comorbidities, predispose to a pathophysiological state characterized by atherosclerosis, dysregulated neuroendocrine modulation, and mechanical hemodynamic abnormalities, thereby constituting a high-risk population for hypertension (Oliveros et al., 2020; Onuh and Qiu, 2020). Consequently, the clinical coexistence of hypertension and HL has become a highly interconnected and intertwined frontier in interdisciplinary research. In routine clinical audiological diagnosis, HL is typically classified by mechanistic features into conductive HL, sensorineural hearing loss (SNHL), and mixed HL (Cunningham and Tucci, 2017). In addition, from a neuro-otological anatomical perspective, HL can be further categorized into PHL–involving peripheral auditory structures from the external ear, middle ear, and inner ear (cochlea) to the auditory nerve prior to entry into the brainstem–and CHL-+, which involves central circuits from the auditory pathway and brainstem to the cerebral cortex. At present, there is insufficient evidence to establish a direct causal relationship between hypertension and CHL; however, based on shared pathological features, long-standing hypertension may lead to small-artery arteriosclerosis and hyaline degeneration, thereby causing chronic hypoperfusion and ischemia (Hainsworth et al., 2024). This process is particularly likely to damage deep white matter, because auditory neural pathways and brain regions involved in complex sound processing rely extensively on white-matter fiber connectivity (Javad et al., 2014). Moreover, hypertension is a major risk factor for both ischemic and hemorrhagic stroke; if stroke involves key nodes of the auditory pathway (such as the auditory cortex, temporal lobe, or auditory nuclei within the brainstem), it can directly result in acute or subacute HL. Hypertension is also an important risk factor for vascular cognitive impairment and dementia and can impair cognitive function in older adults via vascular mechanisms, thereby indirectly reducing the brain’s capacity to process auditory information (Luo et al., 2019).

Clinically, the more common and age-related type is PHL, with SNHL being particularly typical; its occurrence is generally associated with vascular lesions, viral infection, ototoxic medications, systemic inflammation, age-related labyrinthine membrane degeneration, and noise-induced hearing loss (NIHL; Förster et al., 2025). Compared with CHL, the pathological linkage between PHL and hypertension is more direct and more consistently observed, with the key pathogenic locus often considered to be the microcirculation of the inner ear (cochlea). Previous studies have suggested that hypertension may precipitate inner-ear hemorrhage via vascular territories supplied by the anterior inferior cerebellar artery; this artery continues as the internal auditory artery and, together with the cochlear and vestibular arteries, provides perfusion to the inner ear, and hemorrhage in this region may cause progressive or sudden HL (Tsuzuki and Wasano, 2024). In addition, disruption of cochlear oxygen homeostasis can trigger oxidative stress, leading to mitochondrial DNA (mtDNA) damage and functional decline, and may further induce apoptosis of cochlear cells (Yang et al., 2024). Przewoźny et al. (2015) proposed that the stria vascularis, which provides trophic support to the organ of Corti, may represent a key target of hypertension-induced cochlear injury (Reuss, 2025); the pathological process may involve reduced cochlear oxygen partial pressure and disturbed potassium ion recycling (Przewoźny et al., 2015), suggesting that strial dysfunction may be an important mechanism underlying hypertension-related cochlear damage. On the other hand, cochlear injury is also closely related to excessive noise exposure; cumulative noise-induced inner-ear injury is an important contributor to age-related HL (Chen et al., 2021; Hickman et al., 2021). The resulting high-frequency HL may be associated with hypertension (Chang et al., 2011), although heterogeneity across studies should be noted, partly due to factors such as sex.

Building upon prior theoretical frameworks and evidence, this study, for the first time, conducted a systematic, subtype-stratified genetic intersection analysis of hypertension with PHL and CHL. Previous findings suggest substantial overlap among these conditions in mechanisms including hypoxic stress responses, regulation of blood circulation, responses to peptide hormone and humoral signaling, and ion transport regulation. Among these, key pathways–such as AGE-RAGE, PI3K-AKT, cGMP-PKG, and Fluid shear stress and atherosclerosis–jointly constitute a synergistic signaling network that drives metabolic inflammation, mechanical stimulation, and cellular survival and apoptotic processes, thereby linking systemic circulatory dysfunction with progressive functional impairment of the inner ear and central auditory pathways. Hypertension with HL represents a systemic pathological process involving multiple genes and pathways. The shared core genes and key signaling pathways do not operate in isolation but instead constitute a dynamically interacting network. The cascade of events within this network ultimately leads to impaired cochlear microcirculation, exacerbated inflammation and oxidative stress, and auditory cell death.

The AGE-RAGE axis may act as an initiating and amplifying component of the “metabolic–inflammatory–oxidative stress” vicious cycle in this comorbid mechanism: when hypertension is accompanied by metabolic abnormalities (e.g., diabetes), increased accumulation of advanced glycation end products (AGEs) allows AGEs to bind the RAGE receptor and sustain activation of the NF-κB pathway, thereby promoting the release of pro-inflammatory mediators such as tumor necrosis factor-α (TNF-α), interleukin-6 (IL-6), and interleukin-1β (IL-1β), inducing chronic inflammation (Aldea-Perona et al., 2024), and activating intracellular NADPH oxidase to generate excessive reactive oxygen species (ROS; Wautier et al., 2001). The EPIC-Potsdam cohort study found that AGE accumulation was associated with indices of arterial stiffness and may contribute to vascular aging related to aging and hypertension (Birukov et al., 2021). Subsequently, activation of AGE–RAGE and inflammatory signaling, such as TNF, MMP9 expression and activity are markedly increased (Zaremba-Czogalla et al., 2018); excessive activation can disrupt the cochlear basilar membrane and perivascular extracellular matrix, compromise the blood–labyrinth barrier (Wu et al., 2017), promote inflammatory cell infiltration, and may mediate injury to spiral ligament fibroblasts (Xie et al., 2018), ultimately driving persistent alterations in the mechanical properties and microenvironmental homeostasis of the inner ear. In addition, RAGE and AGE scavenger receptors have been identified in the mammalian cochlea (Hanusek et al., 2010). Treatment with asiaticoside can attenuate AGEs/RAGE/NF-κB signaling, enhance antioxidant capacity, reduce hair cell damage, and alleviate hearing threshold elevation (Xing et al., 2017).

Activation of Akt within the PI3K–Akt signaling pathway inhibits proapoptotic proteins, including Bad and Bax, and regulates downstream molecules, including mTOR and FoxO, thereby promoting cell survival and metabolism. In vascular endothelial cells, PI3K activates Akt, which subsequently phosphorylates eNOS and increases NO production, thereby promoting vasodilation (Lee et al., 2018). However, under hypertensive conditions, oxidative stress and inflammatory mediators may suppress the activity of this pathway (Liu et al., 2024). Under the combined effects of oxidative stress, hypoxia- and inflammation-associated DNA damage, and weakened AKT1 survival signaling, TP53 is stabilized and activated (Cao et al., 2024; Dong et al., 2024); activated TP53, as a transcription factor, can upregulate pro-apoptotic genes and further lead to activation of CASP3 (Galichanin et al., 2012). In the mature cochlea, PI3K–Akt signaling generally functions as an endogenous survival pathway in hair cells and supporting cells. Noise exposure that induces a permanent threshold shift has been shown to reduce the expression of the PI3K regulatory subunit p85α and the phosphorylation of Akt at Ser473 in cochlear hair cells and supporting cells. Akt1 knockout increases susceptibility to lower-intensity noise, resulting in more pronounced hearing threshold shifts and hair cell damage (Chen et al., 2015). In addition, cochlear protection is partially dependent on PI3K–Akt signaling, which suppresses mitochondrial apoptosis and caspase-related signaling and maintains the survival of damaged hair cells (Hayashi et al., 2013).

Notably, the fluid shear stress and atherosclerosis pathway integrates the endothelial mechanosensory complex mediated by PECAM1, VE-cadherin, and VEGFR2, which regulates integrin activation, cytoskeletal rearrangement, and endothelial cell alignment in the direction of blood flow (Tzima et al., 2005). Abnormal blood flow patterns induced by hypertension, together with factors such as endothelin-1 (ET-1) secretion within the vascular wall, may induce endothelial dysfunction and early atherosclerosis-like lesions in systemic medium- and large-sized arteries and the spiral artery at the cochlear entrance, whereas inadequate cochlear perfusion may directly impair auditory perception (Kostov, 2021).

The cGMP–PKG axis is an important contributor to vascular dysfunction and the failure of cytoprotective mechanisms within the pathological cascade network linking hypertension and HL. Mice with systemic Npr1 knockout simultaneously exhibit hypertension and auditory abnormalities, providing the closest available evidence for a comorbidity model; however, this model cannot distinguish whether HL arises from systemic hypertension or local cochlear GC-A deficiency. This study demonstrated that cGMP signaling helps attenuate HL resulting from hair cell damage in aging or injured auditory structures (Marchetta et al., 2020). Nitric oxide (NO) released by endothelial cells activates soluble guanylate cyclase (sGC), leading to the production of cyclic guanosine monophosphate (cGMP) and subsequent activation of protein kinase G (PKG). By reducing intracellular Ca^2+^ concentrations and the sensitivity of contractile proteins to Ca^2+^, PKG induces resistance vessel dilation and decreases peripheral vascular resistance (Degjoni et al., 2022). Under hypertensive conditions, reduced endothelial NO bioavailability caused by abnormal shear stress and oxidative stress may directly attenuate cGMP–PKG signaling, predisposing cochlear arterioles to sustained vasoconstriction, exacerbating microcirculatory ischemia, and ultimately leading to PHL. An experimental study found that modulation of the cGMP–PKG axis promoted neurite outgrowth and the survival of cochlear spiral ganglion neurons, suggesting its potential as an effective intervention for inner ear protection (Sun et al., 2021).

Future studies should prioritize establishing routine hearing screening systems and high-precision risk biomarkers; integrate multi-omics approaches to identify ototoxic susceptibility markers to guide precision pharmacotherapy; and strengthen multidisciplinary collaboration to develop clinical and public health strategies encompassing noise protection, individualized medication, and comorbidity management, thereby controlling noise-induced HL and safeguarding hearing health and quality of life in older adults. Based on visualized bibliometric analyses, researchers can rapidly capture research foci and developmental trends in hypertension and HL across domains such as cardiovascular disease and geriatric medicine. Nevertheless, this study has limitations. First, although cross-gene and pathway analyses suggest potential shared mechanisms between the two conditions, robust longitudinal evidence is still lacking to establish causality. Moreover, the identified shared genes may not be specific to hypertension or HL but may instead reflect broader biological pathways associated with aging and inflammation. Therefore, these genes may represent a shared molecular background underlying age-related hypertension and age-related HL rather than disease-specific genes directly linking hypertension to HL. Second, more rigorous study designs and larger sample sizes are needed to delineate the relative contributions of hypertension to PHL versus CHL, particularly in older populations and across different sensory-neural subtypes. Third, bibliometric indicators cannot fully distinguish heterogeneity in study quality; highly productive authors may publish lower-impact work, whereas pivotal breakthroughs by less productive authors may be insufficiently captured. Finally, restricting inclusion to English-language literature may omit important research from non-English-speaking countries, thereby affecting the global representativeness of the conclusions. Future studies should consider incorporating multilingual databases to enhance the comprehensiveness and generalizability of the analysis. A complete direct causal chain underlying the comorbidity of hypertension and HL has not yet been established. This study summarizes a potentially shared molecular network centered on vascular endothelial dysfunction, aberrant mechanosensation, oxidative stress, and inflammatory responses, providing a reference for future investigations into its role in hypertension–HL comorbidity.

## 5 Conclusion

By integrating bibliometric analyses of multi-source databases with genetic intersection analyses, this study systematically evaluated the evolutionary trends, potential interactions, and putative biological mechanisms of hypertension-HL research from 2005 to 2025, highlighting the importance of hypertension in maintaining auditory health. At the mechanistic level, pathways related to AGE-RAGE, PI3K-Akt, cGMP-PKG, and fluid shear stress constitute a cascade pathological network extending from systemic circulatory abnormalities to auditory system–specific injury, and, to some extent, link peripheral (inner-ear) and central (auditory pathway) HL. This network cooperates with key genes including IL6, TNF, TP53, MMP9, EGFR, AKT1, and BCL2 to perturb hemodynamic and metabolic homeostasis, thereby driving a multilayered interaction system centered on inflammation and oxidative stress, with vascular dysfunction and hypoxic stress as pivotal hubs, and culminating in an imbalance between cellular survival and apoptosis. Overall, these lines of evidence support the notion that HL not only constitutes an important component of hypertension-related target-organ damage, but may also, to some extent, participate in the dynamic course of hypertension. In light of current research frontiers, future work should prioritize large-scale prospective clinical studies and high-quality randomized controlled trials to further dissect key nodes and actionable pathways within this interaction network, thereby facilitating effective translation of mechanistic evidence into clinical practice. Meanwhile, bibliometric trends suggest that bridging the translational gap between basic and clinical research has become a consensus in this field; accordingly, it is necessary to establish early screening systems, develop dynamic and precise biomarkers, and explore integrated strategies encompassing diagnosis, treatment, and prognostic assessment, in order to enhance translational impact and provide empirical evidence for comorbidity management and health promotion in the global older population.

## Data Availability

The raw data supporting the conclusions of this article will be made available by the authors, without undue reservation.

## Footnotes

1
https://www.ncbi.nlm.nih.gov/geo/

2
https://www.genecards.org/
