# Supplementary material for: Trends and mechanistic insights into hypertension-associated hearing loss: a 20-year perspective (2005–2025)
Source: Front Mol Neurosci. 2026 Sep 8;19:1864657. doi: 10.3389/fnmol.2026.1864657 (PMC13593908; doi:10.3389/fnmol.2026.1864657)
Supplement: Supplementary file 1 [file Table_1.doc]

Supplementary Material

Figure S1. Enrichment analyses of hypertension- and HL-associated genes in the GEO database. (A) KEGG enrichment analysis of disease-associated genes. (B) GO enrichment analysis of disease-associated genes. (C) DO enrichment analysis of disease-associated genes. Abbreviations: KEGG, Kyoto Encyclopedia of Genes and Genomes; GO, Gene Ontology; DO, Disease Ontology.

Figure S2. Integrated intersection-gene analysis of PHL and Hypertension. (A) Intersection of disease-associated genes between the two conditions. (B) Threshold analysis for filtering intersection genes. (C) KEGG enrichment analysis of intersection genes. (D) GO enrichment analysis of intersection genes. (E) DO enrichment analysis of intersection genes. Abbreviations: KEGG: Kyoto Encyclopedia of Genes and Genomes; GO: Gene Ontology; DO: Disease Ontology.

Figure S3. Integrated intersection-gene analysis of CHL and Hypertension. (A) Intersection of disease-associated genes between the two conditions. (B) Threshold analysis for filtering intersection genes. (C) KEGG enrichment analysis of intersection genes. (D) GO enrichment analysis of intersection genes. (E) DO enrichment analysis of intersection genes. Abbreviations: KEGG: Kyoto Encyclopedia of Genes and Genomes; GO: Gene Ontology; DO: Disease Ontology.
